# The information value of early career productivity in mathematics: a ROC analysis of prediction errors in bibliometricly informed decision making

**DOI:** 10.1007/s11192-016-2097-9

**Published:** 2016-08-08

**Authors:** Jonas Lindahl, Rickard Danell

**Affiliations:** Inforsk (Department of sociology), Umeå University, 901 87 Umeå, Sweden

**Keywords:** Receiver operating characteristic, ROC, Performance, Bibliometric indicator, Prediction errors, Decision making, Productivity, Mathematics

## Abstract

The aim of this study was to provide a framework to evaluate bibliometric indicators as decision support tools from a decision making perspective and to examine the information value of early career publication rate as a predictor of future productivity. We used ROC analysis to evaluate a bibliometric indicator as a tool for binary decision making. The dataset consisted of 451 early career researchers in the mathematical sub-field of number theory. We investigated the effect of three different definitions of top performance groups—top 10, top 25, and top 50 %; the consequences of using different thresholds in the prediction models; and the added prediction value of information on early career research collaboration and publications in prestige journals. We conclude that early career performance productivity has an information value in all tested decision scenarios, but future performance is more predictable if the definition of a high performance group is more exclusive. Estimated optimal decision thresholds using the Youden index indicated that the top 10 % decision scenario should use 7 articles, the top 25 % scenario should use 7 articles, and the top 50 % should use 5 articles to minimize prediction errors. A comparative analysis between the decision thresholds provided by the Youden index which take consequences into consideration and a method commonly used in evaluative bibliometrics which do not take consequences into consideration when determining decision thresholds, indicated that differences are trivial for the top 25 and the 50 % groups. However, a statistically significant difference between the methods was found for the top 10 % group. Information on early career collaboration and publication strategies did not add any prediction value to the bibliometric indicator publication rate in any of the models. The key contributions of this research is the focus on consequences in terms of prediction errors and the notion of transforming uncertainty into risk when we are choosing decision thresholds in bibliometricly informed decision making. The significance of our results are discussed from the point of view of a science policy and management.

## Introduction

Active research policy strategies, especially policies that emphasize excellence, need to develop models for evaluation to prioritize and concentrate limited resources. In such models bibliometric indicators are increasingly used as tools for identify universities, research groups, and researchers that can be said to be “excellent”, and allocating research funding (Whitley and Gläser 2007; Benner 2008; Abramo et al. 2013). However, if we use publication statistics to inform decisions concerning employment and allocate research funds, our main interest should not be to reward past achievement, but rather to enable future scientific achievements. It follows that the use of bibliometric indicators as tools to support an active research policy must be based on the assumption that a researcher’s track record can be used to predict the researcher’s future achievements (Danell 2011; Penner et al. 2013).

The predictive power of bibliometric indicators have been tested in previous research (e.g., Jensen et al. 2009; Danell 2011; Penner et al. 2013; Havemann and Larsen 2015). However, from a decision making perspective it is important to gain knowledge, not only of the degree of correlation/association/relationship between past and future research performance as measured by bibliometric indicators, but also of the potential consequences in terms of costs and benefits of using bibliometric indicators as decision support tools in specific decision scenarios (Penner et al. 2013).

In this article we examine the information value of early career productivity (i.e., publication rate) as a predictor of future productivity from a decision making perspective. Our dataset consisted of 451 early career researchers in the mathematical sub-field of number theory. The purpose was twofold: (1) to provide an analytical framework that can be used to examine and evaluate bibliometric indicators as decision support tools; and (2) to gain knowledge of the potential consequences, in terms of costs and benefits, of using early career productivity as a predictor of future productivity in number theory from the point of view of decision making. We focus on productivity since productivity is an important dimension of research performance that can be quantified with bibliometric indicators (Costas and Noyons 2013). It is a list of publications that examiners are confronted with when making their decision concerning employment or funding, and it has been shown that productivity is the best predictor for career advancement in academia (see e.g., Long et al. 1993). van Arensbergen (2014) show the importance of productivity in the grant allocation process, especially in the early phases of the selection process. An analysis of prediction errors (i.e., costs and benefits) is particularly interesting in the early career phase since events such as a denied or approved application in the early career phase can have long lasting consequences to the career trajectory due to processes of cumulative advantage (Petersen et al. 2011, 2012).

Deciding to approve an application or employ a postdoc are all binary decisions, and to make our research design more similar to actual decision making we treat prediction of future performance as a binary classification problem, i.e. the prediction task was to classify researchers as members or non-members in a future top performance group. In order to classify a researcher as members of a top performing group it is necessary to determine a decision threshold, i.e. some minimum level of early career productivity required to be classified as a top performer. Researchers with a productivity above the decision threshold are considered top performers. However, in order optimize the choice of decision threshold we need to know the consequences of each decision threshold and chose the threshold with the best consequences according to the preferences of the decision maker.

## Research questions

If we want predict who will belong to a future top performance group on the basis of past productivity, we need to find a definition of a top performance group. There is no standard for defining a top performer in terms of productivity in the literature (Costas and Noyons 2013). In this study we used three different definitions of a top performance group. A more inclusive, a more exclusive, and a definition in between the inclusive and exclusive definitions. The use of more than one definition is motivated by the lack of given standards which makes it interesting to compare the consequences of using different definitions. Research question one can be formulated as:

1. How does the definition of the performance groups affect prediction accuracy and prediction errors (i.e., costs and benefits)?

We also compare two methods for determining decision thresholds in a selection process. One method based on the assumption that if a researcher belong in a top performance group, e.g. the top 10 % group, in a past time period, he or she will belong to that top performance group in a future time period as well. We define this method as the simple method. In the second method the decision thresholds are based on a prediction model where consequences are analyzed in order to determine an “optimal” decision threshold. Our second research question can be formulated as:

2. Is there a difference, in terms of prediction errors (i.e., costs and benefits), between decision thresholds that are determined by the simple method, and decision thresholds that are optimized with a prediction model that take the cost of prediction errors into consideration?

Early career productivity can be affected by factors such as collaboration and publication strategies. This motivates an examination of the consequences of adding predictors to the prediction models. Our third research question can be formulated as:

3. How do publications in prestige journals and collaboration early in the career affect prediction accuracy and prediction errors (i.e., costs and benefits)?

## Method

### Data collection

Our data was collected from the MathSciNet (MSN) database, a comprehensive mathematics database with a global coverage provided by American Mathematical Society. The MSN database has two features that make it suitable for bibliometric analysis at the individual level. First, the problem with author name ambiguities (Smalheiser and Torvik 2009) is to a large extent solved in the MSN database for documents published 1985 or later (American Mathematical Society 2014). Second, all articles in MSN are classified according to the Mathematics Subject Classification (MSC) scheme by professional indexers. The MSC classes can be used to delineate sub-fields in mathematics (Dubois et al. 2014).

The main publication channel for mathematical research is peer reviewed journals rather than proceedings, book chapters, or books (American Mathematical Society 2015a). MSN provide three document types: books, journals, and proceedings. Since our aim was to examine research productivity we only included documents indexed as the document type, journals, in our dataset. The final dataset consisted of the journal publication (from now on article) track records of 451 authors in the mathematical sub-field number theory. The authors were identified and selected on the basis of (1) at least one published article in the MCS class 11 (i.e., Number theory) between the years 1999 and 2003; (2) an article publication career of at least 12 years; and (3) that the share of articles belonging to the MSC class in the track record of an author was equal to, or larger, then the share of any other MSC class found in that author’s track record (Costas and Noyons 2013).

Mathematics is a discipline with some features that make it an interesting object of analysis in contexts of predicting research productivity at the individual level. Mathematicians, and especially number theorists, are not dependent on expensive equipment and other resources to conduct research (Dubois et al. 2014). Thus, access to external resources is generally not an important factor for research productivity in mathematics (American Mathematical Society 2015b).

In mathematics the publication volumes are relatively small compared to other fields (American Mathematical Society 2015a). Mathematicians usually write papers as single authors or in small teams (Dubois et al. 2014). Considering our dataset, 33 % of the articles are single authored, 40 % have two authors, 19 % have three authors, and 8 % of the articles have four or more authors. The praxis in mathematics require that all co-authors of a paper has contributed equally to the research (American Mathematical Society 2015c). The praxis of equal author contribution, the focus on individual talent, and the low resource dependence in mathematics, arguably provide an interesting opportunity to investigate productivity at the individual level in a discipline where knowledge production is individually driven, in comparison with other disciplines where productivity, to a higher extent, may be driven by collaboration (e.g., chemistry; medicine).

Another favorable feature of mathematics is that the rate of change over time in terms of knowledge production and formal scientific communication is generally quite slow (Behrens and Luksch 2011). Arguably, this stability increase the generalizability of our findings across time periods.

### Research design and variables

The research design cover two time periods: period 1 and period 2. Period 1 is the first 5 years in the publication career of an author. Period 2 is the eight to the twelfth year. The publication career of a researcher begins with the year of the first article that is indexed in the MSN database.

The response variable was binary and denoted membership or non-membership in a top performance group in period 2. Membership or non-membership in a top performance group is determined by some specified level of article publication rate during period 2. We constructed three different definitions of a top performance group, top 50, top 25, and top 10 %. The threshold for the top 50 % group in period 2 (i.e., between year eight and 12) was the 50th percentile, the threshold for the top 25 % group in period 2 was the 75th percentile, and the threshold for top 10 % group was the 90th percentile. We used the publication output of the 451 authors in period 2 as a reference set to calculate the percentiles (Costas and Noyons 2013). See Table 1 for descriptive statistics of the publication rate in period 1 and period 2.

**Table 1:** Descriptive statistics for publication rate in period 1 (PR P1) and 2 (PR P2), and the two covariates: Publications in prestige journals in period 1 (PPJ) and early career collaboration in period 1 (ECC)

Statistics	PR P1	PR P2	PPJ	ECC
Mean	5.47	5.93	1.93	0.72
1st quartile	3	2	0	0.20
Median	4	4	1	0.63
3rd quartile	7	8	3	1
Min	1	0	0	0
Max	39	46	12	4.33
Sum	2467	2674	–	–

The main predictor consisted of the number of journal articles in period 1: Publication Rate (coded as PR; see Table 1 for descriptive statistics). In addition to the main predictor we created two covariates that could be added to the univariate ROC analysis.

Abramo et al. (2010) showed that researchers in mathematics and computer science with a high publication rate tend to publish in journals with a higher prestige than researchers with a lower publication rate. We find it interesting to test whether the above stated results translates to our research design and our research question concerning the added prediction value of information on publications in prestige journals. Thus, we hypothesize that the inclusion of a journal prestige covariate in the univariate ROC analysis will increase prediction accuracy and decrease prediction errors.

In this study we use the citation based indicator source normalized impact per paper (SNIP), to delineate prestige journal publications in mathematics. We downloaded an excel file from CWTS Journal Indicators that contained a list of all journals indexed in the Scopus database between 1999 and 2014 (CWTS 2015). The CWTS Journal Indicators list contained the journal name with corresponding print-ISSN, e-ISSN, and SNIP-values for each year. Each article in our dataset was matched on the basis of print-ISSN, e-ISSN and full journal title against the journal list provided by CWTS Journal Indicators to obtain a SNIP value. The SNIP values are calculated on the basis of the revised SNIP indicator (Waltman et al. 2012).

A prestige journal was defined as a journal with a SNIP value ≥ the 75th percentile based on a ranking of all journals included in the CWTS Journal Indicators list (CWTS 2015). We calculated one percentile for each year (1999–2014). The early career journal prestige covariate consisted of the number of articles published in journals with a SNIP value equal to or above the 75th percentile in the publication year of the article in period 1: Publications in Prestige Journals (coded as PPJ; see Table 1 for descriptive statistics).

The second covariate address early career collaboration. Collaboration is often controlled for in evaluative bibliometrics. Hu et al. (2014) found a weak negative correlation between collaboration (i.e., the average number of authors per paper) and productivity (i.e., publication rate) in the early career phase in mathematics. The association between early career collaboration and future publication rate in the sub-field number theory is to our knowledge unknown. Although we expect the effect to be small we hypothesize that the inclusion of collaboration covariate in the univariate ROC analysis will increase prediction accuracy and decrease prediction errors. The early career collaboration covariate consists of the average number of co-authorships per publication during period 1: Early Career Collaboration (coded as ECC; see Table 1 for descriptive statistics).

### Data analysis

#### Univariate ROC analysis

The basic model used in the univariate ROC analysis is the confusion matrix (Table 2). The confusion matrix is a cross tabulation of a test outcome versus an actual state (Fawcett 2006). In this study the test outcome is determined by some level of publication rate during the first five years (period 1) in number theory. If a researcher belongs to the actual performance group is determined by some specified level (i.e., a publication rate ≥ the 90, 75, or 50th percentile) of article publication rate between the eighth and the twelfth year (period 2) of the publication career.

**Table 2:** The confusion matrix

		Belong to a specific performance group in period 2	
		True	False
Test outcome	Positive	TP	FP
	Negative	FN	TN

*TP* true positive, *FP* false positive, *FN* false negative, *TN* true negative

Cross tabulation of the test outcome versus the actual state classifies the population into four categories. Researchers for which the test indicated a positive value can either be classified as true positives (TP) if they belong to the specified performance group, or false positives (FP) if they do not belong to the specified performance group. Individuals for which the test outcome is negative can either be classified as false negatives (FN) if they belong to the specified performance group, or as true negatives (TN) if they do not belong to the specified performance group (Fawcett 2006). There are two types of errors in binary prediction models: false positive errors and false negative errors. A perfect prediction model has no false positive errors and no false negative errors (Pepe 2003).

The confusion matrix can be used to calculate several metrics that are important for examining the information value of a prediction model in terms of prediction accuracy and prediction errors. The true positive rate (TPR) is defined as TPR = TP/(TP + FN). The true positive rate denotes the fraction of researchers that are correctly classified as members in a specific top performance group and can be interpreted as an indication of the benefits of a binary prediction model. Synonyms for true positive rate is e.g., sensitivity, recall, hit rate and true positive fraction. The false positive rate (FPR) is defined as FPR = FP/(FP + TN). The false positive rate denotes the fraction of researchers that are misclassified as belonging to a specific top performance group, and can be interpreted as the costs of a binary prediction model. False positive rate is equivalent to the fall-out measure or false positive fraction, and are sometimes defined as 1-specificity. Specificity is equivalent to the true negative rate (TNR). The true negative rate is defined as TNR = TN/(TN + FP). True negative rate denotes the fraction of researchers that are correctly classified as not belonging in a specific top performance group. The false negative rate (FNR) is defined as FNR = FN/(FN + TP) and denotes the fraction of researchers that are misclassified as not belonging to a specific top performance group. The false negative rate is also known as miss rate. The metrics, true positive rate, false positive rate, true negative rate, false negative rate, are actually not rates, but probabilities or fractions (Pepe 2003). These four metrics can take values on the interval [0, 1].

The true positive rate can be interpreted as the conditional probability of being classified as positive given that one belongs to the top performance group (i.e. P(Positive|True)), and false positive rate can be interpreted as the conditional probability of being classified as positive given that one do not belong to the top performance group (i.e. P(Positive|False)). In an examination of the information value of early career publication rate as a predictor for future productivity it is also interesting to ask: What is the conditional probability for making a correct decision given that the test is positive (i.e. P(True|Positive)), and the conditional probability of making a correct decision when the test is negative (i.e. P(False|Negative))? These questions can be answered with the metrics positive predictive value (PPV) and negative predictive value (NPV). Positive predictive value can be defined as PPV = TP/(TP + FP). Negative predictive value can be defined as NPV = TN/(TN + FN). Positive predictive value and negative predictive value measure how well the test result predict the performance level of a researcher. Positive predictive value denotes the fraction correctly classified researchers of all researchers that tested Positive and negative predictive value denotes the fraction correctly classified researchers of all researchers that tested Negative. Positive predictive value and negative predictive value contain values between [0, 1]. On the contrary to true positive rate and false positive rate, predictive values are sensitive to skewed classes in the binary dependent variable (Pepe 2003).

**Fig. 1:**
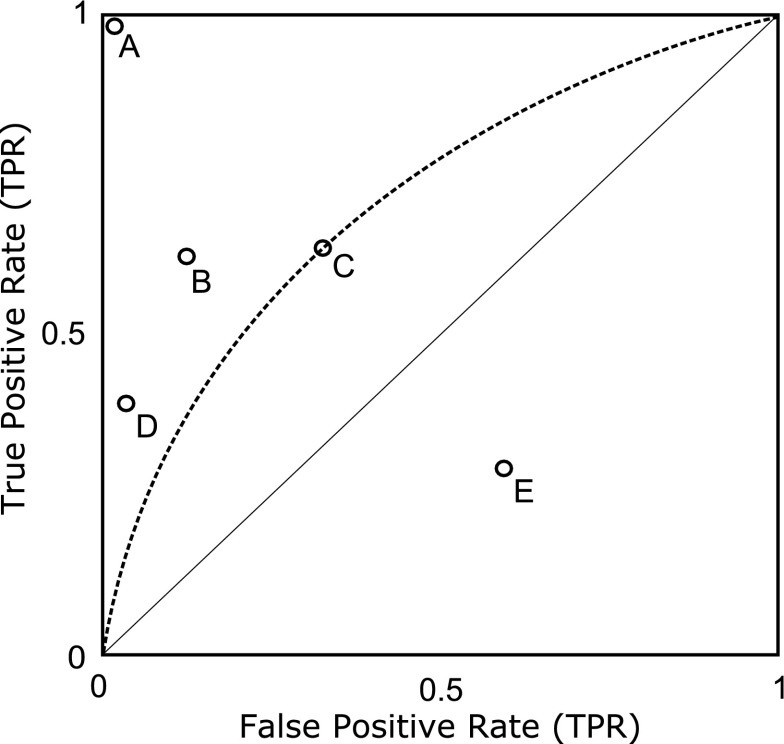
A ROC graph exemplifying five discrete prediction models (**a**, **b**, **c**, **d**, **e**), one ROC curve (*dashed line*), and the reference line (*thin diagonal line*). Adapted from Fawcett (2006)

The ROC graph consists of a two-dimensional ROC space generated by the trade-off between the false positive rate (i.e., costs) and the true positive rate (i.e., benefits) of a binary prediction model (Fawcett 2006). The false positive rate are indicated by the x-axis and the true positive rate are indicated by the y-axis (Fig. 1). Discrete prediction models where the class membership for each unit is defined as either Positive or Negative produce a single confusion matrix (see Table 1). If we were to extract the false positive rate and true positive rate from such a confusion matrix and plot the false positive rate against the true positive rate the result would be one point in ROC space (Fawcett 2006). In Fig. 1a–e, represents the trade-off between the false positive rate and true positive rate for five discrete prediction models.

The position of a point in ROC space represent particular features of the prediction model (Fawcett 2006). Some key positions are important for interpretation. The lower left point (0, 0) in ROC space represent a prediction model that classify all units as Negative (i.e., true positive rate = 0; false positive rate = 0). A prediction model positioned in the upper right point (1, 1) represent a prediction model that classify all units as Positive (i.e., true positive rate = 1, false positive rate = 1). Generally a point in ROC space is considered better if it is positioned closer to the upper left corner (i.e., the true positive rate is higher, the false positive rate is lower, or a combination of higher true positive rate and lower false positive rate). In Fig. 1, b and c have similar true positive rates. However, b has lower false positive rate and can be considered the better prediction model of the two. A perfect prediction model is represented by the point (0, 1). In Fig. 1, a represents a perfect prediction model. Prediction models that are positioned closer to the point (0, 0) on the left hand side of the ROC graph can be considered more exclusive (i.e., the threshold to classify a unit as Positive is high). Prediction models that are positioned closer to the point (1, 1) on the right hand side of the ROC graph can be considered more inclusive (i.e., the threshold to classify a unit as Positive is low). To exemplify, in Fig. 1 the prediction model d is more exclusive than the prediction model c.

It is meaningful to partition ROC space in the positive diagonal. We define this line as the reference line (Fig. 1). If the prediction model is positioned above the reference line it performs better than expected according to a random model. In Fig. 1a–d represents prediction models that perform better than random. If the test variable has a false positive rate and a true positive rate below the reference line (see e in Fig. 1) it performs worse than a random model (Fawcett 2006).

Many prediction models produce an estimate of a unit’s class membership as a probability, or a classification score, to which different thresholds may be applied to predict class membership (Fawcett 2006). For such probabilistic or classification score-based prediction models each cut-off threshold produce a discrete (i.e., binary) prediction model that yields a confusion matrix by which false positive rate and true positive rate can be extracted and plotted as one point in ROC space.

A ROC curve is generated by plotting the range of trade-offs between false positive rate and true positive rate that can be achieved by a given predictor (e.g., publication rate) or prediction model. Conceptually, a ROC curve is generated in ROC space if we produce a confusion matrix, calculate the false positive rate and true positive rate, and plot the false positive rate against the true positive rate for every possible threshold of the probabilistic or classification score-based prediction model (Fawcett 2006). The dashed line in Fig. 1 represents a ROC curve. The representation should be viewed as succession of single points connected by a dashed line. In Fig. 1, c represents one point in the ROC curve. Since the ROC curve is based on the whole range of possible cut-off thresholds, it can provide a more complete description of the performance of a prediction model than metrics from only one cut-off threshold (Pepe 2003).

A commonly used measure to summarize the performance of a ROC curve in terms of prediction accuracy is the area under the ROC curve (Fawcett 2006). The values of the area under the ROC curve (AUC) are between [0, 1]. The area under the ROC curve is 1 when the ROC curve passes through the (0, 1) point (i.e., perfect classification). If the ROC curve coincides with the reference line the area under the ROC curve is 0.5. With an area under the ROC curve less than 0.5 the decision maker is better of flipping a coin. In the context of this study the area under the ROC curve of a prediction model is equal to the probability that a randomly chosen researcher that is classified as positive has a higher value on the test variable (i.e., a higher publication rate; a higher predicted probability) than a randomly chosen researcher that is classified as negative (Fawcett 2006).

When we are using publication track records to inform a decision making process we need to choose a cut-off value (i.e., decision threshold) of the test variable (i.e., publication rate during P1) that can be used as selection criteria so that each individual in the population can be classified as a member or non-member of a future top performance group. Generally the decision threshold is chosen on the basis of the acceptable trade-off between the false positive rate (i.e., researchers that are falsely classified as top performers) and the true positive rate (i.e., researchers that are correctly classified as top performers) given the circumstances of the decision situation (Pepe 2003).

In this study we compare two methods for determining a cut-off threshold on the test variable:

1. Method one is based on the assumption that if a researcher belongs in the top performance group in the first period he or she will belong in the top group in the second period as well. We define this as the simple method since we are using information solely from period 1 without taking the cost of prediction errors into consideration when determining the cut-off thresholds. The simple method represent the common practice when bibliometric indicators are used as decision support tools in science policy and management (see e.g., Coleman et al. 2012; El Emam et al. 2012; Costas and Noyons 2013). The simple method cut-off thresholds consist of percentiles indicating membership in a top 10 % (i.e., 90th percentile), top 25 % (i.e., 75th percentile), and top 50 % (i.e., 50th percentile) performance group in period 1 for predicting membership in a performance group in in period 2. We used the publication output of the 451 authors in period 1 as a reference set to calculate the percentiles (Costas and Noyons 2013). A metric that summarizes the simple method cut-off as a single number is calculated by taking TPR-FPR. Method one is from now on referred to as the Simple method.
2. With the second method we take the cost of prediction error into consideration when determining the cut-off thresholds. A common approach to determine the decision threshold for binary prediction models while taking the cost of prediction error into consideration is to operationalize some definition of an optimal cut-off threshold (Krzanowski and Hand 2009). In this study we define an optimal cut-off value on the test variable as a value that classifies the most number of authors correctly and the least number authors incorrectly given that the true positive rate and false positive rate are equally weighted (Perkins and Schisterman 2006). A metric corresponding to such a definition of an optimal cut-off is the Youden index (Youden index = max(TPR-FPR)) that consist of values on the interval [0, 1], where the maximized difference between the true positive rate and false positive rate over all cut-points is defined as the optimal cut-off value (Perkins and Schisterman 2006). Method two is from now on referred to as the Optimal method. Further, from now on we refer to all cut-off thresholds as decision thresholds.

#### Incorporating covariates into ROC analysis with multiple logistic regression analysis

Factors such as collaboration and publication strategies may influence early career publication rate. It is therefore interesting to evaluate the incremental value of adding covariates univariate ROC models in terms of prediction accuracy and prediction errors (i.e., costs and benefits).

One approach to examine the incremental value of an added covariate in terms of classification accuracy is to fit two logistic regression models, one model with the added covariate and one without the covariate, and create ROC curves on the basis of the predicted probabilities from each model (Janes et al., 2009). Formally the procedure suggested by Janes et al. (2009) is conducted by fitting one logistic regression model with the main predictor or predictors, *X*, and the new covariate, *Y*, and one model without *Y*:

1 $$\text{ln}\left( {OR} \right) = \beta_{0 \, } + \beta_{1} X + \beta_{2} Y$$

and

2 $$\text{ln}\left( {OR} \right) = \beta_{0 } + \beta_{1} X$$

ln(*OR*) denote the natural logarithm of the odds ratio of a positive outcome in Eqs. (1) and (2). In the next step ROC curves are calculated on the basis of the estimated predicted probabilities for all researchers. The predicted probabilities are used to predict class membership for each author (Janes et al. 2009). Finally the two ROC curves based on Eqs. (1) and (2) are compared visually and/or on the basis of some suitable metrics (e.g., the area under the ROC curve).

## Results and discussion

The results and discussion section consists of two parts. In the first part we present and discuss the univariate ROC analysis. In the second part we add covariates to the univariate ROC models with multiple logistic regression analysis. The results are presented as hypothetical decision scenarios. Each definition of a top performance group comprise one of the three decision scenarios: “Decision scenario: Top 10 %”; “Decision scenario: Top 25 %”; and “Decision scenario: Top 50 %”. To exemplify, in the “Decision scenario: Top 10 %”, we imagine, in a broad sense, a performance based system where the top 10 % group is prioritized in contexts of e.g., hiring, promotion and funding in academia. In the “Decision scenario: Top 25 %”, the top 25 % group is prioritized and so on.

Thus, in the univariate ROC analysis we examine the consequences (in terms of prediction errors) of selecting for a particular top performance group in period 2 on the basis the bibliometric indicator publication rate in period 1 given different decision thresholds. In the second part we examine if and how prediction accuracy and prediction errors (i.e., costs and benefits) are affected by adding covariates to the univariate ROC models in the three decision scenarios.

### Univariate ROC analysis of prediction errors in binary decision scenarios

**Fig. 2:**
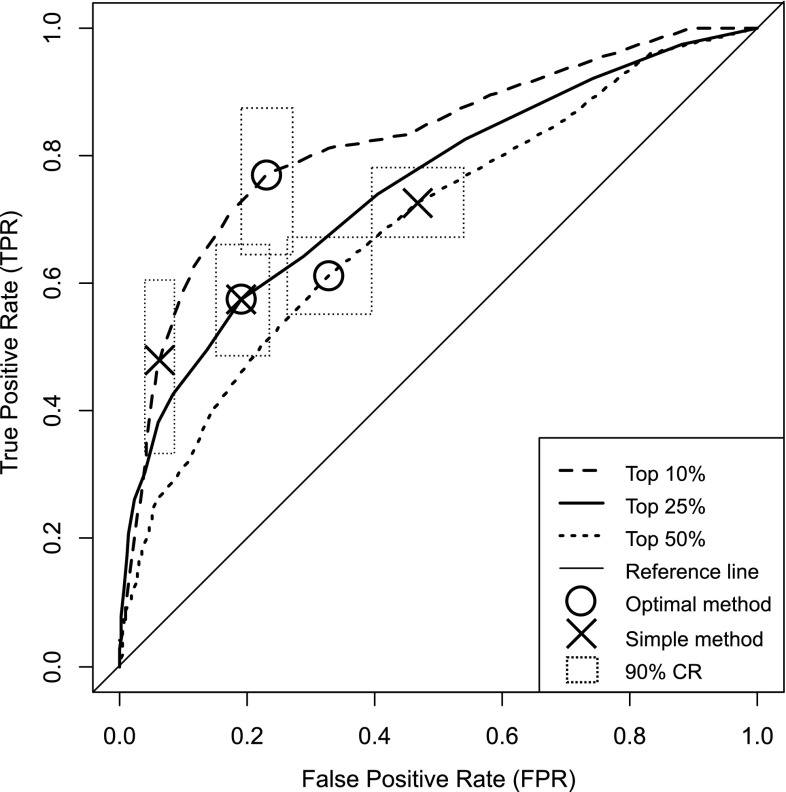
ROC graph representing the trade-off between the true positive rate and the false positive rate for the univariate models. Optimal method = Youden index decision thresholds; Simple method = simple method decision thresholds; 90 % CR = 90 % confidence regions

Figure 2 displays three ROC curves. One ROC curve for each top performance group. Since all ROC curves are well above the reference line it is clear that early career publication rate can be used as an indicator of future publication rate in number theory.

In the decision scenarios we used the observed values to compare different metrics, decision thresholds, and definitions of top performance groups. However, to get an indication of the stability of these observed values, if we were to repeat the analyses a large number of times, we used bootstrap resampling to estimate confidence intervals for the area under the ROC curves, the true positive rate and false positive rate of the decision thresholds, and the positive predictive values and negative predictive values (Robin et al. 2011).

We calculated 95 % confidence intervals (CI) for the area under the ROC curve of each ROC curve (Table 3). The confidence intervals for the area under the ROC curves were computed with bootstrap resampling (percentile method with 2000 stratified bootstrap replicates; Robin et al. 2011).

**Table 3:** The area under the ROC curve (AUC) for the univariate ROC models and 95 % bootstrapped confidence intervals (CI)

Metric	Top 50 %	Top 25 %	Top 10 %
AUC	0.69	0.75	0.82
95 % CI	0.64, 0.74	0.70, 0.80	0.75, 0.89

In Fig. 2 the optimal decision thresholds based on the Youden index (YI) are represented as circles and crosses represent decision thresholds for the Simple method. We calculated a confidence region (CR) for each decision threshold to get an indication of the stability of the observed differences between the Optimal method decision thresholds and the Simple method decision thresholds (Fig. 2; Table 4). The confidence regions for the decision thresholds in the ROC graph (Fig. 2) were computed with bootstrap resampling (2000 stratified bootstrap replicates; Robin et al. 2011) in accordance with the averaging method suggested by Fawcett (2006). Each decision threshold has 95 % confidence intervals in the *x* (i.e., false positive rate) and *y* (i.e., true positive rate) directions (Fawcett 2006). The 95 % confidence intervals for the false positive rate and true positive rate result in a rectangular CR with a 90 % (= 95 % × 95 %) confidence level for both the false positive rate and the true positive rate parameters (Pepe 2003).

**Table 4:** Metrics for decision thresholds derived by the Simple method and the Optimal method

Metric	Simple method	Optimal method
Top 50 %		
TPR-FPR	0.26	–
YI	–	0.29
DT coords: FPR, TPR	0.47, 0.73	0.33, 0.61
90 % CR	0.39, 0.53 × 0.67, 0.78	0.26, 0.39 × 0.55, 0.67
Nr of articles at DT	4	5
Top 25 %		
TPR-FPR	0.38	–
YI	–	0.38
DT coords: FPR, TPR	0.19, 0.57	0.19, 0.57
90 % CR	0.15, 0.24 × 0.49, 0.66	0.15, 0.24 × 0.49, 0.66
Nr of articles at DT	7	7
Top 50 %		
TPR-FPR	0.42	–
YI	–	0.54
DT coords: FPR, TPR	0.06, 0.48	0.23, 0.77
90 % CR	0.04, 0.08 × 0.55, 0.67	0.19, 0.27 × 0.64, 0.88
Nr of articles at DT	11	7

*TPR* true positive rate, *FPR* false positive rate, *YI* Youden index, *DT* decision threshold, *coords* coordinates, *CR* confidence regions, *NR* number

In addition to the 95 % confidence intervals for the area under the ROC curves, and the 95 % confidence region for the Optimal method and Simple method decision threshold coordinates, we calculated 95 % confidence regions for the positive predictive values and the negative predictive values at the Optimal method decision thresholds and the Simple method decision thresholds (Table 5). The 95 % confidence intervals for the positive predictive values and negative predictive values were calculated with bootstrap resampling (percentile method with 2000 stratified bootstrap replicates; Robin et al. 2011).

**Table 5:** Predictive values at decision thresholds for the Simple method and Optimal method

Metric	Simple method	Optimal method
Top 50 %		
PPV at TPR-FPR	0.67	–
PPV at YI	–	0.71
95 % CI	0.63, 0.71	0.67, 0.76
NPV at TPR-FPR	0.60	–
NPV at YI	–	0.57
95 % CI	0.54, 0.66	0.52, 0.62
Top 25 %		
PPV at TPR-FPR	0.51	–
PPV at YI	–	0.51
95 % CI	0.44, 0.58	0.44, 0.58
NPV at TPR-FPR	0.85	–
NPV at YI	–	0.85
95 % CI	0.82, 0.88	0.82, 0.88
Top 10 %		
PPV at TPR-FPR	0.48	–
PPV at YI	–	0.28
95 % CI	0.37, 0.60	0.24, 0.33
NPV at TPR-FPR	0.94	–
NPV at YI	–	0.97
95 % CI	0.92, 0.95	0.95, 0.98

*PPV* positive predictive value, *NPV* negative predictive value, *CI* confidence interval

### Decision scenario: top 50 %

The ROC curve (Fig. 2) in the top 50 % decision scenario had the lowest area under the ROC curve (of the three scenarios) at 0.69 (Table 3). The Youden index for early career publication rate was 0.29 (5 articles) when selecting future members belonging to the Top 50 % group (Table 4). This indicates that in a decision scenario where we would optimize the selection criteria according to the Optimal method (i.e., 5 articles in period 1) for the top 50 % group, 61 % of the future top 50 % performers would be correctly predicted as members of the top 50 % group, and 39 % of the future top performers would be incorrectly classified as non-top performers and therefore excluded in the selection process. A false positive rate at 0.33 indicate that 33 % of the non-top performers would be selected as top performers. As a consequence 33 % of the selected authors would have a publication rate below the top performance percentile threshold in period 2 and thus lowering the overall productivity in the top group.

The decision threshold determined by the Simple method to select for the top 50 % group had a TPR–FPR at 0.26 (4 articles). The true positive rate was 0.73, and the false positive rate was 0.47 (Table 4). In comparison with the Optimal method decision threshold at 5 articles the difference between the two methods for deciding cut-off threshold seem to be trivial. This indicate that the performance level required to belong to the top 50 % group in period 1 as defined by the Simple method, is a good approximation of the Optimal method decision threshold as defined by the Youden index.

The positive predictive value was 0.71 (95 % CI, 0.67, 0.76) at the Optimal method decision threshold for the top 50 % group (Table 5). This indicates that a researcher with a track record of at least 5 articles in period 1 (which is the decision threshold to be classified as a member) would have a probability of 0.71 to actually belong to the top group in top 50 % decision scenario. The negative predictive value was 0.57 (95 % CI, 0.52, 0.62), which indicate that if an author has a publication rate below 5 articles she or he has a probability of 0.57 to actually be a non-member of the top 50 % group. The positive predictive value for the Simple method decision threshold was 0.67 (95 % CI, 0.63, 0.71). The negative predictive value was 0.60 (95 % CI, 0.54, 0.66). Thus, the difference between the Simple method and the Optimal method based on the Youden index seem to be trivial regarding the predictive values as well.

### Decision scenario: top 25 %

With an area under the ROC curve at 0.75 (Table 3), the top 25 % was positioned in between the top 50 % and the top 10 % groups. The Youden index for early career publication rate was 0.38 (7 articles) for the top 25 % group (Table 4). At this decision threshold the true positive rate for the top 25 % group was somewhat lower than for the top 50 % group at 0.57. The false positive rate was higher at 0.19. A false positive rate at 0.19 indicate that 19 % of the authors that are not future top 25 % performers would be incorrectly predicted as top performers in the top 25 % scenario. In a decision scenario where we would use the Optimal method decision threshold (i.e., 7 articles in period 1) for the top 25 % group, 57 % of the future top 25 % performers would be correctly predicted to actually belong to the top 25 % group. The false negative rate was 0.43 (= 1 – 0.57) indicating that 43 % of the actual top performers would be excluded in the selection process when we are selecting for the top 25 % group on the basis of early career publication rate.

The Simple method decision threshold produced similar results as the Optimal method decision threshold, with a TPR-FPR at 0.38 (7 articles) the true positive rate was 0.57, and the false positive rate was 0.19. The 90 % confidence region is identical as can be seen in Fig. 2 and Table 4. The difference between the Simple method and Optimal method seem to be trivial when we are selection for the top 25 % group.

At the Optimal method decision threshold the positive predictive value was 0.51 (95 % CI, 0.44, 0.58, see Table 5). If an author has a track record of at least 7 articles in period 1 (which is the decision threshold to be classified as a member of the top 25 % group), that author has a 51 % chance to actually belong to the top 25 % group. The negative predictive value was 0.85 (95 % CI, 0.82, 0.88), indicating that if a researcher has less than 7 articles the probability of being a non-top performer is 0.85. In comparison with the top 50 % decision scenario the positive predictive value was lower and the negative predictive value was higher when selecting for the top 25 % performance group. The positive predictive value and negative predictive value for the simple method decision threshold was identical with the Optimal method decision threshold in the top 25 % decision scenario.

### Decision scenario: top 10 %

The top 10 % group has the highest area under the ROC curve value at 0.82 (Table 3). This indicate that the indicator of early career publication rate performs best when it is used to predict who will belong to the top 10 % group. However, these results should be interpreted with some caution since the confidence intervals for the area under the ROC curve is overlapping for all but the top 10 and 50 % groups. The Youden index for the Optimal method was 0.54 (7 articles) for the top 10 % group with a true positive rate at 0.77 (Table 4). If we would optimize the decision threshold according to the Optimal method for the top 10 % group (i.e., 7 articles in period 1), 77 % of the future top 10 % performers would be correctly predicted as top performers. The false negative rate was 0.23 (= 1 – 0.77) indicating that 23 % of the top performers would be missed in the selection process in the top 10 % decision scenario. While the optimal decision threshold for selecting top 10 % performers had the highest true positive rate of the three scenarios, the false positive rate was higher compared to the top 25 % scenario and lower compared to the top 50 % scenario at 0.23.

The Simple method decision threshold had a lower TPR-FPR at 0.42 (compared to the Youden index for the Optimal method at 0.54), and a more exclusive decision threshold of 11 articles compared to the Optimal method decision threshold at 7 articles (Table 4). Further, the true positive rate was 21 % lower for the Simple method compared to the true positive rate at the Optimal method decision threshold. The 90 % confidence region for the Optimal method and the Simple method decision thresholds derived is clearly not overlapping (Fig. 2). This indicate that the difference is statistically significant between the Optimal method decision threshold and the Simple method decision threshold in the top 10 % decision scenario.

While the Simple method seem to provide a good approximation for the Optimal method decision thresholds when selecting for the top 25 and 50 % groups, there was a significant difference between the two methods when selecting for the top 10 % group. The Simple method resulted in a more exclusive (i.e., high) decision threshold and the Optimal method resulted in a more inclusive (i.e., low) decision threshold. A consequence of an inclusive decision threshold, compared to an exclusive threshold, is that more individuals are included in the selection. Thus, if we would use the Optimal method in a selection process where the top 10 % group is prioritized we would get less prediction errors compared to the Simple method. However, the inclusion of more individuals may be viewed as a cost that must be balanced against the benefit of reducing prediction errors.

If we turn to the Simple method, we see that the true positive rate was very low and thus the false negative rate was very high, and as a consequence the Simple method would miss a large fraction of potential future top performing mathematicians (see Fig. 2; Table 4). False negative prediction errors may be particularly problematic early in the career because the outcome of events such as a funding decision or a job application may have long lasting consequences to the career trajectory (see e.g., Long et al. 1979; Petersen et al. 2012). However, in decision scenarios where the cost of a false positive decision is very high (e.g., a tenure track position; Penner et al. 2013), an exclusive decision threshold may be preferable even if it leads to a high false negative rate. We conclude that the choice of method and decision thresholds depends on how the decision maker values the cost of different prediction errors.

As can be seen in Table 5 the positive predictive value for the Optimal method decision threshold was 0.28 (95 % CI, 0.24, 0.33) for the top 10 % group (i.e., with the information that an author has a publication track record of at least 7 articles a decision maker would know that the chance of picking a future top performer is 28 percent). Thus, even though the area under the ROC curve and the true positive rate was relatively high, the positive predictive value was relatively low at 0.28. The reason for the low positive predictive value is likely a consequence of the skewed classes in the dependent variable, since predictive values are dependent on the prevalence of top performers (Pepe 2003). When selecting for the 50 % group, on the other hand, where the classes are less skewed the true positive rate was 0.61, with 71 percent of the selected individuals actually belonging to the top performance group according to the positive predictive value. The negative predictive value was 0.97 (95 % CI, 0.95, 0.98), indicating that if we know that a researcher have a publication rate below 7 in period 1, there is a 97 % chance that he or she actually is a non-top performer. (Tables 4, 5).

The positive predictive value for the Simple method decision threshold was 0.48 (95 % CI, 0.37, 0.60). Naturally the positive predictive value increase in a scenario where we use a more exclusive decision threshold (Table 5). Compared to the Youden index threshold at 7 articles, the Simple method threshold at 11 articles increased the probability top group membership given a positive test with 20 %. In accordance with the Optimal method decision threshold the negative predictive value was high at 0.94 (95 % CI, 0.92, 0.95).

We end this section with a discussion of results from the univariate ROC analysis. First, the analysis of the area under the ROC curve showed that it is easier to predict future productivity if the performance group is defined as the top 10 %, than it is to make predictions if the top performance group is defined as Top 25 % or Top 50 %. This result indicates that the information value of the predictor publication rate differ depending on how top performance is defined. Danell (2011) found similar results in the context of predicting who will write highly cited papers. If we wish to develop models to prioritize and concentrate resources on the basis of bibliometric indicators this result indicate that it may be important to take the definition of top performer or top performance group into consideration when estimating these models. A good prediction model where we are selecting for top 10 % performers may not work as well for top 50 % performers. However, since the confidence intervals were overlapping for all but the top 50 and top 10 % performance groups, these findings should be interpreted with some caution.

Second, the difference between the Simple method and the Optimal method suggest that the method by which the decision thresholds are determined may matter when bibliometric indicators are used as decision support in, e.g., processes of grant selection or staff selection. Our results also suggest that the Optimal method may provide better decision support in some cases, compared to the Simple method which is the method commonly used in practice (see e.g., Coleman et al. 2012; El Emam et al. 2012; Costas and Noyons 2013). We conclude that the usefulness of a method to determine the decision threshold depends on the context and how the costs of different prediction errors are assessed. In the context of science policy there has been a growing need to estimate the costs and benefits of different choices (in terms of e.g., financial, societal, or scientific discoveries; Lane et al. 2011). The costs and benefits of using bibliometric indicators as decision support tools has to our knowledge gained little attention in the literature. In this study we have defined costs as prediction errors. An investigation on how we can assess and assign actual costs, such as economic, social, or scientific costs, to prediction errors in the context of bibliometricly informed decision making could potentially increase the efficiency of using bibliometric indicators as decision support tools in academia. However, such an investigation is beyond the scope of this study.

Third, even if the Simple method and the Optimal method produce similar results, there is an important conceptual difference between the methods that we believe is important to highlight. The Simple method rest on the assumption that decision thresholds based on information of scholarly performance in the past is good enough to provide useful decision support, given the preferred outcome (e.g., to enhance research performance; to increase scholarly progress in general, to increase productivity). However, this assumption may not hold true. To asses whether or not a decision threshold actually is useful we need information on consequences. With the Optimal method we are analyzing the consequences of all decision thresholds in terms of prediction errors and can chose the decision threshold that is optimal according to the preferred outcome. The difference between the two methods can be represented by two concepts from decision theory: uncertainty and risk. A decision maker acts under uncertainty when the consequences of a decision is unknown (March and Heath 1994; Knight 2007). It is very difficult to make well informed decisions under uncertainty. Under conditions of risk, on the other hand, the probabilities with which the consequences of a decision may occur is known to the decision maker. The main difference between decision making under uncertainty and risk, is the amount of information that is available to the decision maker (March 1994; Knight 2007). The Simple method is equivalent to decision making under uncertainty. However, when we are analyzing the consequences of different decision thresholds in terms of prediction errors (e.g., as suggested by the Optimal method) we are transforming uncertainty into risk. According to Moed (2007) it is the task of the bibliometric community to provide information of the risks of using bibliometric methods and indicators in the evaluation process, and the task of the scholarly community as a whole and the domain of science policy to assess these risks and decide whether the benefits outweigh the costs. One key contribution of this study is that it extends the line of research concerned with prediction of scientific performance at the individual level (see e.g., Jensen et al. 2009; Danell 2011; Penner et al. 2013; Havemann and Larsen 2015) to binary decision making and the transformation of uncertainty into risk when we are choosing decision thresholds.

#### Evaluating the incremental value of adding covariate information to the univariate ROC models

We examined the incremental value of adding covariates to the univariate ROC model in terms of prediction accuracy following the approach suggested by Janes et al. (2009). We fitted one multiple logistic regression model for each decision scenario consisting of the main predictor Publication Rate (i.e., PR) and the covariate Publications in Prestige Journals (i.e., PPJ, defined as the total publication output in period 1 in journals with a SNIP value ≥ the 75th percentile) according to Eq. (1). We also fitted one model for each decision scenario consisting of the main predictor and the covariate Early Career Collaboration (i.e., ECC) according to Eq. (1). For each of these six models the area under the ROC curve was calculated on the basis of the predicted probabilities from the logistic regression together with 95 % bootstrapped confidence intervals (percentile method with 2000 stratified bootstrapped replicates). Each bootstrap replicate comprised the whole procedure including random sampling with replacement from the 451 authors, estimating the logistic regression model, extracting the predicted probabilities from the logistic regression, calculating the ROC curve and estimating the area under the ROC curve (Janes et al. 2009).

We compared the area under the ROC curve from the multiple logistic regressions with the area under the ROC curve values from the univariate ROC analysis (Table 6). We estimated logistic regression models for the univariate models (i.e., with only the covariate Publication Rate) as well to obtain Pseudo-R2.

**Table 6:** The area under the ROC curve (AUC), 95 % confidence intervals (CI), and Pseudo-R2 for nine logistic regression models

Metric	PR	PR + PPJ	PR + ECC
Top 10 %			
AUC	0.82	0.82	0.82
95 % CI	0.75, 0.89	0.75, 0.88	0.75, 0.89
Pseudo-R2	0.216	0.221	0.217
Top 25 %			
AUC	0.75	0.75	0.75
95 % CI	0.70, 0.80	0.70, 0.80	0.70, 0.81
Pseudo-R2	0.165	0.166	0.165
Top 50 %			
AUC	0.69	0.70	0.69
95 % CI	0.64, 0.74	0.65, 0.75	0.65, 0.74
Pseudo-R2	0.088	0.095	0.088

*PR* Publication rate, *PPJ* Publications in prestige journals, *ECC* Early career collaboration

Table 6 show the area under the ROC curve and 95 % confidence intervals for the univariate model consisting solely of the Publication Rate covariate (denoted PR in Table 6), the multiple model consisting of the covariates Publication Rate and Publications in Prestige Journals (denoted PR + PPJ in Table 6), and the multiple model with the covariates Publication Rate and Early Career Collaboration (denoted PR + ECC in Table 6). As can be observed the classification accuracy did not improve with the multiple models in any of the decision scenarios. A model (not shown) that included all three covariates showed similar results. Thus, we could not confirm our hypotheses that information on publications in prestige journals or early career collaboration would increase prediction accuracy. Due to the null results we do not carry on with a presentation of the ROC curves and the analysis of decision thresholds and positive and negative predictive values. However, similar to the analysis of the area under the ROC curve, these analyses did not show any significant differences between the multiple and univariate ROC models.

We end this section with a brief discussion of the results from the assessment of the incremental value of adding covariate information to the univariate ROC models. Due to the null results we find the meaningful discussion to revolve around the methodology and the usefulness of the information it may provide in contexts of science policy and management.

An important question for decision makers is how much better the outcome of a decision would be if some new information is taken into consideration. The method of evaluating the incremental value address that question (Janes et al. 2009). Bibliometric evaluation at the individual level should not rely on a single indicator since many factors may influence research performance (Costas and Noyons 2013). To account for the complexity of research performance a series of complementary indicators that measure different dimensions of scientific performance should be used (Moed 2007; Costas and Noyons 2013). This poses the problem of how to effectively and meaningfully combine different indicators (Moed 2007). If we wish to use combinations of bibliometric indicators, e.g., to allocate research funds or for hiring decisions, we need methods to evaluate the added prediction value, decision thresholds, and prediction errors, of individual indicators in combination with other indicators. The method of analyzing the incremental value of adding covariate information to ROC models as presented in this study is one method that can be used for that purpose.

## The effect of varying career length

Lastly, we want to address an issue concerning the selection procedure used to obtain our dataset. Only authors that had active publication careers for at least 12 years were included in the analysis. As a consequence the models show prediction accuracy and prediction errors for authors that remained productive over a long time (i.e., a subset of the initial cohorts). An advantage with this restriction is that all included authors share similar career trajectories (Haslam and Laham 2009). With a publication career length of, e.g., at least 1 year instead of 12 years, it would have been difficult to clearly discern whether we were predicting future publication rate or publication career length. By restricting the publication careers to at least 12 years we avoided that problem.

A disadvantage with a career length of at least 12 years is that the potential effects of researchers leaving academia or stop publish early in the career is not accounted for in the analyses (Haslam and Laham 2009). To get an indication of how the outcome change as the inclusion criteria change we calculated true positive rate and false positive rate for six different career lengths on the basis of the initial cohorts. Table 7 provide the area under the ROC curve given a career length of at least 1, 3, 5, 8, 10, and 12 years for each of the three top performance groups. As can be seen in Table 7 the area under the ROC curve for all groups are highest at a career length of ≥1 year. This is expected since many authors had a track record with only one publication. After a career length cut-off at ≥3 years the area under the ROC curve values seem to stabilize in all three performance groups. This indicate that once the authors with a career length of ≥1 year is excluded from the cohorts, the career length cut-off does not essentially alter the outcome (at least in terms of area under the ROC curve).

**Table 7:** Displaying how the area under the ROC curve (AUC) changes with different career lengths and different definitions of the top performance group

AUC at given career length	Top 50 %	Top 25 %	Top 10 %
AUC, career length ≥1 year	*	0.82	0.85
AUC, career length ≥3 year	0.69	0.72	0.77
AUC, career length ≥5 year	0.69	0.74	0.82
AUC, career length ≥8 year	0.69	0.73	0.81
AUC, career length ≥10 year	0.70	0.75	0.81
AUC, career length ≥12 year	0.69	0.75	0.82

* The 50th percentile had the value 0 at career length ≥1 year. Thus, there was no variation in the binary dependent variable

## Conclusions

The purpose of this study was to present an analytical framework that can be used to examine the information value of early career productivity in a binary decision situation and to investigate the potential consequences, in terms of prediction errors, of using early career productivity to predict future productivity in the mathematical sub field of number theory. In this study we have investigated the consequences of using different thresholds in the prediction model, we have investigated the effect of different definitions of top performance groups—top 10, top 25, and top 50 %—and we have investigated the added prediction value of information on publications in prestige journals early in the career and early career collaboration.

From our analysis of the area under the ROC curve we conclude that early career productivity has an information value in all tested decision scenarios, but future productivity is easier to predict when the performance group is defined as top 10 %, i.e. future productivity is more predictable if the performance group is more exclusive. If we wish to use bibliometric indicators to inform science policy this result indicate that it may be important to take the definition of a top performer or top performance group into consideration when we are assessing these indicators, since a good prediction model when we are selecting for the top 10 % performance group may not work as well for top 50 % group. However, the generalizability of these results need further validation.

When using an indicator such as publication rate it is necessary to decide on a cutoff value that can be used as a decision threshold. In this study we compared two methods for deciding on a decision threshold. One simple and straight forward method based on the assumption that if an individual belongs in the top group in the first period he or she will belong in the top group in the second period. The Simple method represents the common praxis when bibliometric indicators are used as decision support tools and does not take the cost of prediction errors into consideration. The second method, defined as the Optimal method, was based on the Youden index and take the cost of prediction errors into consideration. We conclude that for the top 50 and the top 25 % performance groups both methods gave the same result. For the top 10 % group the Optimal method gave a more inclusive decision threshold value of 7 articles, compared to the 11 articles that was the lower limit in the top 10 % group in the first period. These differences indicate that the method by which we determine the decision thresholds matter when we are using bibliometric indicators as decision support in context of science policy and management. However, the usefulness of a decision threshold depends on how the cost of different prediction errors are assessed. We conclude that the choice of method to determine the decision threshold depends on the decision context.

We investigated the added prediction value of information on publications in prestige journals and collaboration early in the career with multiple logistic regression and ROC analysis. We hypothesized that information on early co-authorship and publication strategies would make the prediction better. We can conclude that neither variables improved the prediction in any of the decision scenarios. However, since many factors may influence research performance (e.g., age, gender, mobility, research environment, etc.) and the praxis in evaluative bibliometrics is to combine several complementary indicators (Moed 2007), we need methods to evaluate the added prediction value of new information in order to take these aspects into consideration when bibliometric indicators are used as decision support tools in academia. The ROC framework can provide such methods.

One of the key contributions of this research is the focus on consequences in terms of prediction errors and the notion of transforming uncertainty into risk when we are choosing decision thresholds. A future venue of research could revolve around the question of how to assign actual costs (e.g., economic or social) to prediction errors in the context of bibliometricly informed decision making. The generalizability of our results may extend to other mathematical sub-fields oriented towards pure mathematics. Another useful line of research could be to apply the ROC framework to other fields with different publication practices compared to mathematics, such as physics, chemistry or medicine, where productivity to a larger extent is driven by, e.g., access to resources and collaboration. It would also be interesting to extend the ROC framework to other career phases, other dimensions of research performance and examine the added prediction value of covariates other than early career collaboration and publications in prestige journals.
